# Use of a Modified Preexposure Prophylaxis Vaccination Schedule to Prevent Human Rabies: Recommendations of the Advisory Committee on Immunization Practices — United States, 2022

**DOI:** 10.15585/mmwr.mm7118a2

**Published:** 2022-05-06

**Authors:** Agam K. Rao, Deborah Briggs, Susan M. Moore, Florence Whitehill, Doug Campos-Outcalt, Rebecca L. Morgan, Ryan M. Wallace, José R. Romero, Lynn Bahta, Sharon E. Frey, Jesse D. Blanton

**Affiliations:** ^1^National Center for Emerging and Zoonotic Infectious Diseases, CDC; ^2^Kansas State University College of Veterinary Medicine, Manhattan, Kansas; ^3^Epidemic Intelligence Service, CDC; ^4^University of Arizona College of Medicine, Phoenix, Arizona; ^5^Department of Health Research Methods, Evidence and Impact, McMaster University, Hamilton, Ontario; ^6^Arkansas Department of Health; ^7^Minnesota Department of Health; ^8^Saint Louis University School of Medicine, St. Louis, Missouri.

Human rabies is an acute, progressive encephalomyelitis that is nearly always fatal once symptoms begin. Several measures have been implemented to prevent human rabies in the United States, including vaccination of targeted domesticated and wild animals, avoidance of behaviors that might precipitate an exposure (e.g., provoking high-risk animals), awareness of the types of animal contact that require postexposure prophylaxis (PEP), and use of proper personal protective equipment when handling animals or laboratory specimens. PEP is widely available in the United States and highly effective if administered after an exposure occurs. A small subset of persons has a higher level of risk for being exposed to rabies virus than does the general U.S. population; these persons are recommended to receive preexposure prophylaxis (PrEP), a series of human rabies vaccine doses administered before an exposure occurs, in addition to PEP after an exposure. PrEP does not eliminate the need for PEP; however, it does simplify the rabies PEP schedule (i.e., eliminates the need for rabies immunoglobulin and decreases the number of vaccine doses required for PEP). As rabies epidemiology has evolved and vaccine safety and efficacy have improved, Advisory Committee on Immunization Practices (ACIP) recommendations to prevent human rabies have changed. During September 2019–November 2021, the ACIP Rabies Work Group considered updates to the 2008 ACIP recommendations by evaluating newly published data, reviewing frequently asked questions, and identifying barriers to adherence to previous ACIP rabies vaccination recommendations. Topics were presented and discussed during six ACIP meetings. The following modifications to PrEP are summarized in this report: 1) redefined risk categories; 2) fewer vaccine doses in the primary vaccination schedule; 3) flexible options for ensuring long-term protection, or immunogenicity; 4) less frequent or no antibody titer checks for some risk groups; 5) a new minimum rabies antibody titer (0.5 international units [IUs]) per mL); and 6) clinical guidance, including for ensuring effective vaccination of certain special populations.

## Background

Transmission of rabies virus occurs when saliva or neural tissue from an infected mammal is introduced into a person or another animal through, for example, a bite or contact with mucous membranes (*1*). Worldwide, approximately 59,000 human rabies deaths occur each year (*2*). The canine rabies virus variant (CRVV) is the most common source of human rabies infections, accounting for approximately 98% of cases, including some cases among U.S. travelers (*3*). In the United States, CRVV has been eliminated (*3*), but wildlife rabies remains endemic, accounting for approximately 5,000 reported rabid animals each year (*4*). Specific wildlife rabies virus variants (RVVs) associated with mesocarnivores (small to midsized animals whose diet includes 50%–70% meat) are endemic in distinct geographically confined locations in 42 U.S. states and Puerto Rico (*4*). In contrast, bat RVVs are widely distributed throughout the United States, with only Hawaii being rabies-free (*3*). During January 2000–December 2020, 52 cases of human rabies were diagnosed in the United States, 38 of which were indigenously acquired (i.e., from rabies exposures that occurred in the United States) (*4*); none were in persons who had previously received PrEP.

In the United States, two modern cell culture vaccines are licensed for rabies PrEP and PEP: human diploid cell vaccine (HDCV; Imovax/Sanofi Pasteur)* and purified chick embryo cell vaccine (PCECV; RabAvert/Bavarian Nordic),^†^ respectively; both are packaged for intramuscular (IM) administration (*1*). Each IM dose of vaccine consists of 1 mL and should be administered in the deltoid for adults, and in either the deltoid or anterolateral aspect of the thigh for children.

## Reasons for Revisions of Recommendations

ACIP has recommended rabies PrEP since 1969 (*5*). As safe and effective modern cell culture vaccines have replaced those derived from nerve tissue and duck embryo, and as rabies epidemiology has continued to evolve (e.g., elimination of CRVV, emergence and spread of the racoon RVV, and host shifts of bat RVV to mesocarnivores in the southern United States), changes have been made to ACIP recommendations. Since 2008, when the last ACIP rabies PrEP recommendations were published, barriers affecting adherence to the recommendations have been identified, including out-of-pocket costs of rabies biologics (3-dose PrEP vaccination series is currently estimated at ≥$1,100^§^), confusion about which activities fall within different risk categories, and noncompliance with recommendations for repeated titer checks (*6*). In addition, travel medicine providers have indicated that the largest group for which PrEP is recommended (travelers to regions with endemic CRVV) might often be unable to complete the 3-dose series described in the 2008 ACIP recommendations (*1*) because at least 21 days are required to complete the series before initiation of travel (*7*).

During September 2019–November 2021, the ACIP Rabies Work Group participated in monthly or bimonthly teleconferences and considered evidence-based updates to the 2008 ACIP recommendations. The Work Group comprised experts in diverse disciplines including laboratory, public health, and clinical specialties. Data collected, analyzed, and prepared by the Work Group were deliberated by ACIP during six public meetings. With publication of this report, the recommendations become final and are the official CDC recommendations for rabies PrEP.

## Redefined Risk Categories

Recommendations for PrEP depend on the level of a person’s risk for being exposed to rabies. The Work Group redefined risk categories into five groups, with level 1 involving activities with the highest risk and level 5 involving those with the lowest risk (Table). The highest risk categories (levels 1 and 2) include exposures that might be unrecognized (i.e., not perceived by the exposed person); for example, a small scratch to the skin during an inconspicuous personal protective equipment breach might not be noticed by persons testing neural tissue from a rabid animal or conducting ecologic studies on bats in the field. For persons with risk for unrecognized exposures, checking serial titers has historically been advised to ensure maintenance of persistently elevated rabies antibody titers; in its recent discussions, ACIP upheld this guidance because the assumption is that high titers might provide some protection when PEP is not sought for an unrecognized exposure. Recognized exposures, as defined by ACIP, are those bites, scratches, and splashes for which PEP would be sought because the exposures are usually registered by a person as unusual (e.g., contact with a bat) or painful (e.g., bite or scratch from a raccoon). The Work Group concluded that most high-risk activities involving live animals (e.g., providing veterinary health care or participating in outdoor activities in countries with endemic CRVV) are associated with only recognized exposures (risk categories 3 and 4); ACIP concluded that checking serial titers for these persons is unnecessary because recognized exposures should always prompt evaluation for PEP. Rabies vaccination recommendations for each of the redefined risk categories is summarized under Recommendations.

**TABLE:** Rabies preexposure prophylaxis recommendations — United States, 2022

Risk category	Nature of exposure	Typical population*	Relevant disease biogeography^†^	Recommendations	
				Primary PrEP^§^ immunogenicity	Long-term immunogenicity^¶^
1. Elevated risk for unrecognized** and recognized^††^ exposures including unusual or high-risk exposures	Exposure, often in high concentrations, might be recognized or unrecognized, might be unusual (e.g., aerosolized virus)	Persons working with live rabies virus in research or vaccine production facilities or performing testing for rabies in diagnostic laboratories	Laboratory	IM rabies vaccine on days 0 and 7	Check titers every 6 months; booster if titer <0.5 IU/mL^§§^
2. Elevated risk for unrecognized** and recognized^††^ exposures	Exposure typically recognized but could be unrecognized; unusual exposures unlikely	Persons who frequently 1) handle bats, 2) have contact with bats, 3) enter high-density bat environments, or 4) perform animal necropsies (e.g., biologists who frequently enter bat roosts or who collect suspected rabies samples)	All geographic regions where any rabies reservoir is present, both domestic and international	IM rabies vaccine on days 0 and 7	Check titers every 2 years; booster if titer <0.5 IU/mL^§§^
3. Elevated risk for recognized^††^ exposures, sustained risk^¶¶^	Exposure nearly always recognized; risk for recognized exposures higher than that for the general population and duration exceeds 3 years after the primary vaccination	Persons who interact with animals that could be rabid***; occupational or recreational activities that typically involve contact with animals include 1) veterinarians, technicians, animal control officers, and their students or trainees; 2) persons who handle wildlife reservoir species (e.g., wildlife biologists, rehabilitators, and trappers); and 3) spelunkers	All domestic and international geographic regions where any rabies reservoir is present	IM rabies vaccine on days 0 and 7	1) One-time titer check during years 1–3 after 2-dose primary series; booster if titer <0.5 IU/mL,^§§^ or 2) booster no sooner than day 21 and no later than year 3 after 2-dose primary series^†††^
		Selected travelers. PrEP considerations include whether the travelers 1) will be performing occupational or recreational activities that increase risk for exposure to potentially rabid animals (particularly dogs) and 2) might have difficulty getting prompt access to safe PEP (e.g., rural part of a country or far from closest PEP clinic)	International geographic regions with rabies virus reservoirs, particularly where rabies virus is endemic in dog populations		
4. Elevated risk for recognized^††^ exposures, risk not sustained^¶¶^	Exposure nearly always recognized; risk for exposure higher than for general population but expected to be time-limited (≤3 years from the 2-dose primary PrEP vaccination series)	Same as for risk category 3 (above), but risk duration ≤3 years (e.g., short-term volunteer providing hands-on animal care or infrequent traveler with no expected high-risk travel >3 years after PrEP administration)	Same as for risk category 3 (above)	IM rabies vaccine on days 0 and 7	None
5. Low risk for exposure	Exposure uncommon	Typical person living in the United States	Not applicable	None	None

**Abbreviations**: IM = intramuscular; IU = international units; PEP = postexposure prophylaxis; PrEP = preexposure prophylaxis.

* Nature of exposure and type of work performed are the most important variables to consider when determining a person’s risk category. The examples provided are intended to be a guide, but ultimately categorizations should be done on a case-by-case basis with nature of exposure considered. Some persons might be categorized into a different risk group from those suggested by the provided examples. For example, most veterinarians are in risk category 3 because they are at risk for recognized exposures after direct contact with animals. However, a veterinary pathologist who often performs necropsies on mammals suspected to have had rabies might have risk for rabies virus exposure that is more consistent with risk category 2 than risk category 3; such persons should follow the recommendations for the risk category with which their activities best fit. Similarly, most spelunkers do not often enter high-density bat caves; those who do may follow the recommendations for risk category 2 rather than risk category 3. Persons involved in the diagnosis of rabies virus, but for whom the frequency of handling rabies virus–infected tissues is low, or the procedures performed do not involve contact with neural tissue or opening of a suspected rabid animal’s calvarium could consider following the recommendations for risk category 2 rather than those for risk category 1.

^†^ Local or state health departments should be consulted for questions about local disease biogeography.

^§^ Primary immunogenicity refers to immunogenicity that peaks 2–4 weeks after completing the recommended primary vaccination schedule. Persons without altered immunity are expected to mount appropriate responses, and checking titers is not routinely recommended. Persons with altered immunity are advised to confirm, through laboratory testing, a rabies antibody titer ≥0.5 IU/mL ≥1 week after booster vaccination (but ideally, 2–4 weeks after completing the recommended schedule) and before participating in high-risk activities. Individual laboratories set facility-specific rules about whether acceptable antibody titers should be laboratory-confirmed for all personnel, regardless of whether personnel have altered immunity.

^¶^ Long-term immunogenicity refers to the ability to mount an anamnestic response to rabies virus >3 years after completion of the primary rabies vaccination series.

** Unrecognized exposures are those that recipients might not know occurred; for example, a small scratch during an inconspicuous personal protective equipment breach might not be noticed by persons testing neural tissue from a rabid animal or persons conducting ecologic studies on bats in the field.

^††^ Recognized exposures are bites, scratches, and splashes that are usually registered by a person because the exposure is unusual (e.g., contact with a bat) or painful (e.g., bite or scratch from a raccoon).

^§§^ When rabies antibody titers are <0.5 IU/mL, a booster vaccination should be provided. Antibody titers to verify booster response need not be checked after these boosters are administered to persons who are immunocompetent. For persons who are immunocompromised, the indicated antibody titer should be verified ≥1 week (ideally, 2–4 weeks) after administration of every booster vaccination.

^¶¶^ Sustained risk is elevated risk for rabies >3 years after the completion of the primary rabies PrEP vaccination schedule.

*** Rabies virus is unlikely to persist outside a deceased animal’s body for an extended time because of virus inactivation by desiccation, ultraviolet irradiation, and other factors. Risk from transmission to persons handling animal products (e.g., hunters and taxidermists) is unknown but presumed to be low (risk category 5); direct skin contact with saliva and neural tissue of mammals should be avoided regardless of profession.

^†††^ Checking titers after recommended booster doses is not indicated unless the recipient has altered immunity.

Risk categories might change over a person’s lifetime. Some persons for whom PrEP is indicated might have elevated risk for a limited period (e.g., during a summer internship working with wildlife or a month-long vacation to a rural village where CRVV is enzootic [risk category 4]). After the event has passed, risk level and associated recommendations for such persons will change. Shifts in risk categories are explained in the management of deviations from the recommendations section under Clinical Guidance.

## Minimum Acceptable Rabies Antibody Titer Level

A correlate of protection for rabies antibody titers has not been defined. The minimum antibody level historically recommended by ACIP is one that results in complete neutralization of rabies virus at a 1:5 serum dilution by the rapid fluorescent focus inhibition test. This is approximately equivalent to a titer of 0.1–0.3 IU/mL. Stakeholders have advocated for a specific titer value in IU/mL units of measure (rather than a range) and, ideally, one that aligns with current global guidance (i.e., that of the World Health Organization) (*8*). Although no infections among vaccinated persons occurred with the previous ACIP cut-off titer, most published studies use 0.5 IU/mL as a correlate of protection. This level is now endorsed by ACIP and replaces the previous minimum acceptable rabies antibody titer. The higher value provides a more conservative limit for indicating inadequate response to rabies vaccination and the need for booster doses (*9*).

## Evidence for Updated Vaccine Schedule and Recommendations for Booster Doses and Titer Checks

Although there is no established correlate of protection for rabies, induction of a peak antibody response at or above the minimum acceptable antibody titer level (≥0.5 IU/mL) in response to rabies vaccine is an indirect measure of protection (i.e., immunogenicity). Primary immunogenicity refers to immunogenicity that peaks 2–4 weeks after completing the recommended vaccination or vaccinations and elicits an anamnestic response to rabies virus exposures. Since publication of the 2008 ACIP recommendations (*1*), scientists have been evaluating data concerning the efficacy of shorter rabies PrEP dosing regimens.

Subject matter experts performed a systematic review of scientific evidence published during 1965–2019 for a 2-dose primary vaccination series (doses administered on days 0 and 7) compared with the 3-dose series (doses administered on days 0, 7, and 21 or 28), which is indicated in the 2008 ACIP recommendations (*1*). Data showed that an anamnestic response after the 2-dose series occurs at 3 years (*10*); however, an anamnestic response >3 years after the 2-dose series has not been evaluated. In the absence of data confirming an anamnestic response, the Work Group evaluated methods of inferring long-term immunogenicity (i.e., an anamnestic response >3 years after the 2-dose primary vaccination series). Checking a titer or titers was considered one way of inferring long-term immunogenicity as described in the PrEP schedule and long-term immunogenicity section that follows. As an alternative to a titer check, a second systematic review was conducted to evaluate a booster dose after the 2-dose series compared with no booster dose. The Work Group used an adapted Grading of Recommendations Assessment, Development and Evaluation (GRADE) approach to determine the certainty of evidence for immunogenicity rated on a scale of 1 (high certainty) to 4 (very low certainty). Within the evidence to recommendations (EtR) framework, ACIP considered the importance of rabies as a public health problem; the benefits and harms (including GRADE-assessed evidence); the target populations’ values and preferences; and issues of resource use, acceptability to stakeholders, feasibility of implementation, and anticipated impact on health equity.

**PrEP schedule and primary immunogenicity.** The systematic review identified 12 studies that enrolled a combined total of 1,401 subjects. Studies evaluating both IM and intradermal vaccination were included because primary immunogenicity is similar for both routes of administration (*11*). Using the GRADE approach, the Work Group concluded with moderate (level 2) certainty that the primary immunogenicity of the 2-dose (days 0 and 7) IM schedule is comparable to that of the 3-dose (days 0, 7, and 21 or 28) IM schedule (risk ratio = 1.00 [95% CI = 0.99–1.01]).^¶^ ACIP deliberated whether the 2-dose (days 0 and 7) IM PrEP schedule should replace the 3-dose schedule for all persons for whom rabies PrEP is indicated based on this finding and other findings within the EtR framework**: the target population’s acceptability of the 2-dose series, feasibility of implementing the 2-dose series, minimal resource use, and anticipated increase in health equity because the 2-dose series is less expensive than the 3-dose series.

**PrEP schedule and long-term immunogenicity.** Serial antibody titer checks are recommended for persons at elevated risk for unrecognized exposures. During recent discussions, ACIP upheld this recommendation advising that rabies antibody titers be checked every 6 months for persons in risk category 1 and every 2 years for persons in risk category 2. As previously noted, the main reason to maintain high titers is to provide some protection from unrecognized exposures; however, high titers also ensure an anamnestic response after an exposure (i.e., long-term immunogenicity).

For persons at sustained risk for only recognized exposures (risk category 3), checking serial antibody titers (as recommended for risk groups 1 and 2) was determined unnecessary; a one-time check of rabies antibody titer during years 1–3 after the 2-dose primary series was deemed appropriate assurance of long-term immunogenicity for persons with this risk. The rationale for this conclusion is that data indicate that an antibody titer ≥0.5 IU/mL 1 year after a rabies PrEP schedule is a marker for long-term immunogenicity (*12*,*13*), and the 2-dose series is known to be protective for at least 3 years (*10*).

As an alternative to the one-time titer check for risk category 3, the systematic review identified observational data from two studies that showed a booster dose triggered an anamnestic response up to 3 years after the 2-dose series. Because the third dose of the PrEP series recommended in the 2008 ACIP recommendations is given as early as day 21 and is known to provide long-term immunogenicity, a booster dose administered from day 21 to year 3 after the primary series was considered. Using the GRADE methodology, the Work Group concluded with low (level 3) certainty that a one-time booster dose of rabies vaccine during day 21–year 3 after the primary vaccination series provides better long-term immunogenicity than no booster dose; low certainty was determined because the data were not from randomized controlled trials comparing the booster with no booster.^††^ After evaluating these data, ACIP considered an IM booster dose of rabies vaccine during day 21–year 3 after completing the 2-dose series as an alternative to a titer check, for persons with sustained and elevated risk for recognized rabies exposures (i.e., those in risk category 3) from day 21 to year 3 after completing the 2-dose series. The rationale for the recommendation^§§^ within the EtR framework included the public health importance of rabies, moderately substantial desirable anticipated effect from administering a booster dose, minimal anticipated undesirable effects, acceptability to stakeholders, and feasibility of implementing the booster dose.

## Recommendations

After considering the evidence, ACIP recommended all persons for whom rabies PrEP is indicated receive 2 IM doses of HDCV or PCECV on days 0 and 7. In addition, persons in the newly defined risk category 1 should have rabies antibody titers checked every 6 months, and those in the newly defined risk category 2 should have rabies antibody titers checked every 2 years; a booster dose should be administered if titers are <0.5 IU/mL at the time of these title checks (Table). ACIP recommended persons in risk category 3 either have rabies antibody titers checked during years 1–3 after completion of the 2-dose primary series (and a booster dose if the titer is <0.5 IU/mL) or preemptively receive a one-time IM booster dose of rabies vaccine during day 21–year 3 after completion of the 2-dose primary series (Figure). These recommendations apply both to immunocompetent and immunocompromised persons; however, PrEP administered to immunocompromised persons requires additional considerations as described in the approach to PrEP section under the following Clinical Guidance.

**Figure:**
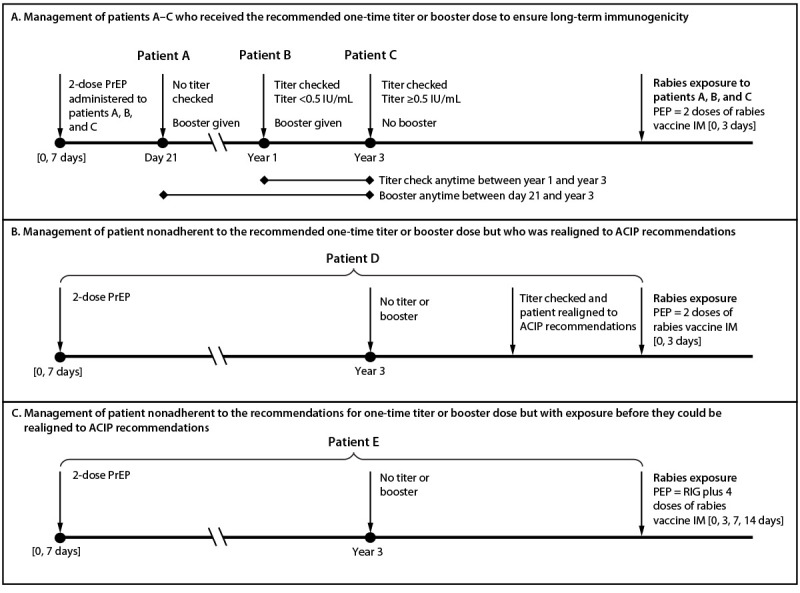
FIGURE. Management of long-term immunogenicity* for hypothetical patients (A–E)†,§,¶ who received the Advisory Committee on Immunization Practices recommended 2-dose rabies preexposure prophylaxis schedule** and have sustained risk for recognized exposures (risk category 3) — Advisory Committee on Immunization Practices, United States, 2022 **Abbreviations:** ACIP = Advisory Committee on Immunization Practices; IM = intramuscular injection; IU = international units; PEP = postexposure prophylaxis; PrEP = preexposure prophylaxis; RIG = rabies immunoglobulin. * Long-term immunogenicity is considered a successful anamnestic response (i.e., rapid rise in antibody levels) after an encounter with the rabies virus antigen >3 years after the primary vaccination series. ^†^ Patient A received the recommended booster dose during day 21–year 3 and patients B and C received the recommended one-time titer check during years 1–3. Recommended options for patients A–C include 1) a one-time rabies vaccine booster dose from day 21 to 3 years after the 2-dose primary series (patient A) and 2) a one-time rabies antibody titer check 1–3 years after the 2-dose primary series (patients B and C). ^§^ Patient D did not receive the recommended one-time titer or booster dose but was realigned to the ACIP recommendations before an exposure occurred. Realigning involves checking a titer. If the titer is ≥0.5 IU/mL, no further action is needed, and the patient is considered realigned with the ACIP recommendations. If the titer is <0.5 IU/mL, patient D should receive a booster dose followed by an additional titer no sooner than 1 week later (preferably 2-4 weeks later) to confirm the appropriate response. ^¶^ Patient E did not receive the recommended one-time titer or booster dose and had an exposure before they could be realigned to the ACIP recommendations. This patient should receive RIG and the 4-dose rabies vaccine PEP series indicated for persons not previously vaccinated. ** An acceptable antibody titer (i.e., ≥0.5 IU/mL) should be confirmed after boosters are administered to immunocompromised persons.

## Clinical Guidance

The Work Group identified additional considerations that are essential to effective administration of rabies PrEP. These include coadministration of PrEP and chloroquine (or drugs related to chloroquine), the approach to PrEP in special populations, and management of deviations from the ACIP recommendations.

**Coadministration of IM rabies PrEP and chloroquine or drugs related to chloroquine**. Recent data show that although concomitant administration of chloroquine and IM rabies PrEP is associated with a significant reduction in rabies antibody titer, the reduced levels remain >0.5 IU/mL (*14*). This finding is of uncertain clinical significance because immunocompetent persons who receive chloroquine and rabies vaccines would presumably mount rabies antibody titer levels ≥0.5 IU/mL and therefore not require management that differs from that for persons who did not receive concomitant rabies vaccine. However, out of an abundance of caution and because rabies is nearly always fatal, clinicians might consider avoiding chloroquine when rabies vaccine is being administered. If avoidance is not possible, ensuring that a patient’s rabies antibody titer is ≥0.5 IU/mL no sooner than 1 week (preferably 2–4 weeks) after completion of the series will confirm that vaccination was effective. No impact on efficacy was observed in the same study when other antimalarials (i.e., Malarone [atovaquone plus proguanil] and doxycycline) were administered with IM rabies PrEP. Limited anecdotal reports suggest mefloquine does not impair rabies vaccine effectiveness (*15*); however, large-scale trials are needed to evaluate this hypothesis.

**Approach to PrEP in special populations, including persons suspected or confirmed to be immunocompromised.** Modern rabies vaccines are inactivated and have been safely administered to persons of all ages, including pregnant women and immunocompromised persons. An adequate immune response is anticipated among all immunocompetent persons (including elderly immunocompetent persons) who receive rabies vaccines in accordance with the ACIP recommendations. For this reason, proof of primary immunogenicity through laboratory confirmation is not advised for immunocompetent persons after the following actions: completion of the 2-dose primary series; administration of booster doses for serial titers <0.5 IU/mL (risk categories 1 and 2) or the one-time titer <0.5 IU/mL (risk category 3); and administration of a one-time booster dose (risk category 3).

However, among persons with primary or secondary immunodeficiencies, the immune response to vaccines, including rabies vaccines, can be suboptimal. ACIP recommends that, when possible, vaccination be delayed until a temporary immunocompromising condition has resolved or immunosuppressive medications can be withheld.^¶¶^ If an immunocompromising condition cannot be temporarily reversed, rabies vaccines can be administered, but antibody titer should be checked no sooner than 1 week (preferably 2–4 weeks) (*10*) after completion of the 2-dose PrEP series and all booster doses (including those administered within 3 years of the primary series and in response to a low titer during the serial titer checks recommended for risk categories 1 and 2). If the titer is <0.5 IU/mL, a booster dose should be administered, followed by a subsequent titer check. If two such booster doses fail to elicit an acceptable antibody titer, local or state public health authorities should be consulted for case-specific guidance. Participation in high-risk activities by persons confirmed or suspected to be immunocompromised should be avoided until the laboratory-confirmed minimum acceptable antibody titer is achieved or until public health authorities provide alternative guidance. Of note, if deviations in the ACIP recommendations occur as described in management of deviation section below, a titer check is recommended regardless of immune status.

**Management of deviations from the recommendations.** Unavoidable delays of a few days from the recommended date of the second dose of the 2-dose primary series are clinically inconsequential. The effect of longer lapses of 2 weeks or more is unknown. When substantial delays occur, local and state public health authorities should be consulted for guidance. The second dose of the primary series should not be administered before the recommended interval between doses has elapsed; if it is inadvertently administered earlier, local and state public health authorities should be consulted for guidance.

Persons who have not previously received rabies PrEP should identify the risk category based on their activities. If their activities change over time, the recommendations of the new risk category should be followed to ensure long-term immunogenicity. Persons in risk category 3 who do not obtain the titer check or booster dose recommended by ACIP within the specified interval can be realigned with the ACIP recommendations (i.e., they should first have a random titer checked regardless of their immune status); for some, titers remain ≥0.5 IU/mL (*16*) and a booster dose is not required. However, for those whose titer is <0.5 IU/mL, a booster should be administered and then titers checked no sooner than 1 week (preferably 2–4 weeks) later. Once a titer of 0.5 IU/mL is achieved, these persons should be managed the same as persons who, consistent with the ACIP recommendations, had the recommended titer or booster within 3 years of the 2-dose primary vaccination series vaccine (Figure).

Persons who have not realigned with the ACIP recommendations and have a rabies exposure require the same PEP that is recommended for persons who did not receive PrEP (i.e., rabies immunoglobulin and 4 IM doses of rabies vaccine on days 0, 3, 7, and 14) (*17*). After this, they are considered to have been previously vaccinated, and in response to any subsequent exposure, only require 2 doses of rabies vaccine on days 0 and 3. Similarly, persons whose risk was categorized as category 4 (e.g., because of short-term animal care work), might later in life shift to risk category 3 (e.g., because they are pursuing a veterinary career). Shifts from risk category 3 to risk category 4 should be managed through realignment with the ACIP recommendations described; if realignment is not done, in response to an exposure to rabies virus should be managed with rabies immunoglobulin and the 4-dose rabies vaccine series (doses administered on days 0, 3, 7, and 14)

## Implications of These Updates

More persons who are recommended to receive rabies PrEP might be vaccinated because the 2-dose series recommended in these updates is associated with lower out-of-pocket costs and takes less time to complete. Persons with only short-term (≤3 years) risk for rabies (risk category 4) require no additional titer or booster doses, and last-minute travelers who previously were not vaccinated because the 3-dose series required ≥21 days might now be vaccinated because only 1 week is needed to complete the 2-dose primary series.

The updates might also facilitate improved adherence to evidence-based ACIP recommendations. As previously mentioned, in the past, some persons recommended to have serial titers checked did not adhere to those recommendations; with this update, many such persons now have the option of a one-time titer check or a one-time booster dose (i.e., a one-time action with two options for accomplishing it). As described in the EtR framework, some persons might prefer the titer option because of the potentially lower cost if a booster is not indicated (i.e., titer is ≥0.5 IU/mL); others might prefer the convenience of proceeding directly to a booster dose. The wide interval during which the titer or booster options can be taken might defray up-front costs and allow persons more time to determine whether they expect risk for rabies >3 years. Appointments for the titer check or booster dose can be scheduled at the time of the 2-dose primary series to ensure adherence to the recommendations.

Persons who received the 3-dose PrEP schedule recommended by ACIP in the past and whose activities place them within risk category 3 require no further titer checks or booster doses; the last vaccine dose they receive as part of the 3-dose series is equivalent to the option provided in these updates for a booster dose during day 21 to year 3. However, frequency of serial titer checks (risk categories 1 and 2) is unchanged, regardless of whether the 2-dose or 3-dose primary series was received by a person.

A consequence of the updated minimum acceptable rabies antibody titer (0.5 IU/mL) is that when titers are checked, more persons might require a booster dose than with the previous minimum acceptable rabies antibody titer level. ACIP concluded that the benefits of the new acceptable titer outweighed this theoretical concern.

## Future Research

Ongoing studies are needed to confirm long-term immunogenicity of the 2-dose PrEP series >3 years after the primary series. Studies are also needed to evaluate the frequency of and need for titer checks for persons in risk categories 1 and 2 and to examine efficacy of PrEP among immunocompromised persons.

**Summary**

**What is already known about this topic?**

Rabies is a zoonotic infection that is nearly always fatal. Preexposure prophylaxis (PrEP) is recommended for certain persons at high risk for exposure.

**What is added by this report?**

During 2019–2021, the Advisory Committee on Immunization Practices made multiple updates to the rabies PrEP recommendations, including the following: a 2-dose (days 0 and 7) intramuscular rabies vaccination series replaced the 3-dose schedule, a one-time titer or booster dose was advised for persons with risk for only recognized rabies exposures, risk categories were redefined, and the minimum acceptable rabies antibody titer was changed to 0.5 IU/mL.

**What are the implications for public health practice?**

The updates are as efficacious as previous recommendations and might facilitate improved adherence to vaccination recommendations.

## Footnotes

*
https://www.fda.gov/media/75709/download

†
https://www.fda.gov/files/vaccines%2C%20blood%20%26%20biologics/published/Package-Insert---RabAvert.pdf

§
https://www.cdc.gov/vaccines/acip/meetings/downloads/slides-2021-02/24-25/03-Rabies-Rao-508.pdf

¶
https://www.cdc.gov/vaccines/acip/recs/grade/rabies-2-dose.html

**
https://www.cdc.gov/vaccines/acip/recs/grade/rabies-2-dose-etr.html

††
https://www.cdc.gov/vaccines/acip/recs/grade/rabies-booster-dose.html

§§
https://www.cdc.gov/vaccines/acip/recs/grade/rabies-booster-dose-etr.html

¶¶
https://www.cdc.gov/vaccines/hcp/acip-recs/general-recs/immunocompetence.html
